# Ambient anthropogenic noise but not light is associated with the ecophysiology of free-living songbird nestlings

**DOI:** 10.1038/s41598-017-02940-5

**Published:** 2017-06-05

**Authors:** Thomas Raap, Rianne Pinxten, Giulia Casasole, Nina Dehnhard, Marcel Eens

**Affiliations:** 1 Department of Biology, Behavioural Ecology and Ecophysiology Group, University of Antwerp, Wilrijk, Belgium; 2 Faculty of Social Sciences, Antwerp School of Education, University of Antwerp, Antwerp, Belgium

## Abstract

Urbanization is associated with dramatic increases in noise and light pollution, which affect animal behaviour, physiology and fitness. However, few studies have examined these stressors simultaneously. Moreover, effects of urbanization during early-life may be detrimental but are largely unknown. In developing great tits (*Parus major*), a frequently-used model species, we determined important indicators of immunity and physiological condition: plasma haptoglobin (Hp) and nitric oxide (NOx) concentration. We also determined fledging mass, an indicator for current health and survival. Associations of ambient noise and light exposure with these indicators were studied. Anthropogenic noise, light and their interaction were unrelated to fledging mass. Nestlings exposed to more noise showed higher plasma levels of Hp but not of NOx. Light was unrelated to Hp and NOx and did not interact with the effect of noise on nestlings’ physiology. Increasing levels of Hp are potentially energy demanding and trade-offs could occur with life-history traits, such as survival. Effects of light pollution on nestlings of a cavity-nesting species appear to be limited. Nonetheless, our results suggest that the urban environment, through noise exposure, may entail important physiological costs for developing organisms.

## Introduction

As a consequence of urbanization, anthropogenic noise and light have dramatically increased over the recent decades and they pose a worldwide environmental challenge^1–6^. Mounting evidence raises concerns about their environmental and health impacts, and a wide variety of behavioural, physiological and fitness effects have been reported (reviewed in ref. 3). While most studies have investigated these pressures in isolation, urbanization is often associated with an increase in both noise and light. It is therefore crucial to study these anthropogenic pressures simultaneously to determine whether they have an additive effect or whether the combined effects are stronger than the sum of their parts (synergistic effect)^3, 7–9^. Such studies are urgently needed for effective mitigation and management of protected areas especially because anthropogenic noise and light may amongst other effects lead to a loss of species and have negative consequences for populations, communities and ecosystems^1, 3, 10, 11^.

Most studies on anthropogenic noise and light have focused on effects in adults in the laboratory (but see e.g. refs 12–15), but experiences during early-life in the wild may profoundly alter individual physiology and health in later life. Environmental conditions experienced during development can shape individual life histories and therefore potentially lifetime reproductive success^16^. Noise exposure can have a major impact on behaviour and physiology^17^. Noise can increase stress^18^, reduce the immune response^19^ and may entail important costs for developing organisms. For example, experimental noise exposure reduced telomere length of free-living house sparrows (*Passer domesticus*) which likely affects their longevity^12^. The immature circadian system may be particularly sensitive to circadian disruption through artificial light and experiences during early-life may have profound negative effects on the developing brain, influence adult behaviour, physiology, health and disease^20^. While studies have shown that noise and light can have negative behavioural and physiological effects on adult birds, results on nestlings are almost completely missing (but see e.g. refs 12–15). These are equally important, especially as early-life experiences will have long-term effects on these individuals.

Therefore, we studied simultaneously the variation in noise and light exposure of free-living great tit (*Parus major*) nestlings in an urban population and related exposure levels to important indicators of short term survival, physiological condition and health: fledging mass, haptoglobin (Hp) and nitric oxide (NOx). Fledging mass is a good proxy for condition as heavier nestlings have higher nutritional reserves^21^, resulting in higher survivorship and recruiting probabilities^22–24^. Haptoglobin plays an important role in inflammation, infection and trauma. It acts as an antioxidant and is part of the non-specific immune response (reviewed in ref. 25). Plasma nitric oxide is a multifunctional signalling molecule and involved in inflammatory processes, although uncontrolled production may lead to cell damage and death (reviewed in ref. 26). Haptoglobin and NOx have also previously been shown to be affected by light at night in an experimental field study^14^. Haptoglobin and NOx may therefore provide useful information on physiological condition, health state and innate immunity^25, 26^. This may generate a better understanding of underlying physiological mechanisms that may link anthropogenic noise and light exposure to potential health and fitness consequences. While in a previous experimental study we have shown that artificial light at night inside the nest box affects body mass gain, Hp and NOx, little is known about how ambient levels of light pollution affect developing great tits. Moreover it is unknown whether noise pollution also has an effect and whether the combined effect of noise and light is additive or synergistic.

We expected negative effects of the combined effect of noise and light pollution. Noise exposure alone appears not to affect fledging mass^12^ but effects on the immune response have been reported^19^. Developing great tits exposed to artificial light at night had increased Hp and decreased NOx levels^14^ and a reduced growth rate^15^. Given that noise and light in songbirds can influence foraging behaviour of parents^27, 28^ and sleep behaviour of nestlings^29^, and noise may impair parent offspring communication^30^, we anticipated a negative impact of noise and light on individual health and condition through direct and/or indirect effects. Streets are often associated with noise and light pollution. For example, in our study population the highway represents the main source of noise pollution. However, roads may have negative effects on animals other than those through noise and/or light pollution^31, 32^. For example, road-related chemical pollution may affect oxidative stress and inflammatory responses (reviewed in ref. 8). Therefore, distance to the nearest road or to the highway was considered as an alternative explanation.

## Methods

### Study site and data sampling

Data were collected during the 2015 breeding season (between 8 and 25 May) in a resident suburban nest box population of great tits in the surroundings of Wilrijk (Antwerp), Belgium (51°9’44”N, 4°24’15”E). This nest box population was established in 1997 and has been continuously monitored since then (e.g. refs 33–37). In order to determine laying date, hatching date and brood size, we checked nest boxes every other day. Nestlings that were 15 days old (hatch day = day 1) were weighed to obtain fledging mass (conform Halfwerk, *et al*.^38^; 0.1 g; digital balance; Kern TCB 200-1) and blood sampled (≤150 μl) from the brachial vein. Blood samples were kept cool and were centrifuged within a few hours after sampling to separate red blood cells from plasma. We did not obtain sufficient amounts of blood from all nestlings in order to perform all analyses, resulting in different sample sizes (fledging mass: 562 nestlings from 85 nests; Hp: 475 nestlings from 78 nests; NOx: 344 nestlings from 58 nests). Sixteen nests from the current study had also been used in a previous experiment as a control group but these were not manipulated (see refs 14 and 15). Nests that were exposed to experimental artificial light inside the nest box during that experiment were excluded from the current study. This study was approved by the ethical committee of the University of Antwerp (ID number 2014-88) and performed in accordance with Belgian and Flemish laws.

Nestlings’ ambient noise and light exposure were measured at each nest box after sunset. In order to minimize disturbance of nestlings and parents, both noise (DVM 401 environmental meter, Velleman Inc., Fort Worth, TX, USA) and light intensity (ILM 1335 light meter, ISO-TECH, Northamptonshire, UK) were measured at the nest box opening. These measurements were taken as a proxy for nestling exposure to anthropogenic noise and light. The main source of light pollution comes from street lights while the main source of noise pollution is from the highway adjacent to the study area. Nightly noise measurements (>1 hour after sunset to 1 hour after midnight) were taken during spring of 2012–2015 at mostly the same nest boxes (Table 1). In 2015 also daytime noise measurements were taken (between 8:30–12:30). We registered the highest value of background noise amplitude, measured during 10 s. Measurements were made only when there was no car passing (except for those on the highway) or other extreme source of noise and therefore measurements represent background noise. As is the case in many other studies^3^, we relied on a relatively simple and inexpensive metric of noise. However, noise measurements were highly correlated among years (Table 2) and between day (*N* = 79) and night time (*N* = 79) measurements in 2015 (Pearson *r* = 0.6, *P* < 0.001) which confirms the reliability of our measurements. Moreover, according to a report by the Flemish government, noise levels in our study area are similar throughout the day and between working days and weekends^39^. Our measurements are also consistent with those taken by the Flemish government, implying that we can be confident that areas with high levels of noise in their report correspond with nest boxes exposed to high noise levels in our study. The main source of noise pollution in our study area, the highway, is one of the busiest highways of Belgium and noise levels are therefore similar throughout the day and among years. Average noise measurements from 2015 were subsequently used as an approximation of the level of noise pollution to which the nestlings were exposed. Light levels ranged between 0.01 and 6.4 lux (0.01 lux is the lower limit of the light meter).

**Table 1:** Average noise levels in our study population. Average noise levels are given per year.

Year	Average noise (dB) ± SE	N
2012	51.5 ± 0.6	69
2013	54.3 ± 0.8	74
2014	53.4 ± 0.8	84
2015	53.1 ± 0.8	79

Sample sizes (N) varied between years but consisted mostly of the same nests, with 67 nests being measured in all four years.

**Table 2:** Noise measurements in our study population are highly correlated over the years.

	2012	2013	2014
**2013**	0.69		
**2014**	0.61	0.66	
**2015**	0.54	0.52	0.57

Spearman rank correlation coefficient (adjusted for multiple tests; Holm correction) are given for nightly noise measurements between 2012–2015. All correlations were significant (*P* < 0.001).

### Sexing, haptoglobin and nitric oxide determination

Following earlier research on great tits, we determined nestling sex (from red blood cells) and quantified Hp and NOx concentrations (from blood plasma)^36, 40, 41^. Nestling sex was determined genetically^42^. Plasma Hp concentrations (µg/ml) were quantified using the manufacturer’s instructions provided with the commercially available colorimetric assay (PHASE Haptoglobin assay, Tridelta Development Ltd)^25^. To quantify NOx concentrations (µmol/l) we used the spectrophotometric assay based on the reduction of nitrate to nitrite by copper-coated cadmium granules^26^. The inter assay coefficient of variability was 4.5% for Hp and 3.9% for NOx.

### Statistical analyses

All statistical analyses were conducted in R 3.1.2^43^. We first tested whether our data exhibited spatial autocorrelation to avoid possible pseudoreplication^44^ which was not the case; models with and without an auto-correlation structure (ratio, spherical, exponential, Gaussian and linear correlation structure) were compared using AIC and inclusion of an auto-correlation structure did not improve the model (AIC increased). We then examined whether light and/or noise (average of day and night time noise measurements in 2015) explained variation in fledging mass, Hp or NOx, by constructing a set of linear mixed models (LMM) for each of these three dependent offspring parameters (lme4 package^45^). Noise and light levels were not correlated with each other (Spearman rank *r* = 0.2, *p* = 0.2, *N* = 85). Nest identity (NestID) was included in all models as random factor to avoid pseudoreplication. The model with fledging mass as dependent variable contained brood size (covariate), laying date (covariate) and noise (covariate), light (log + 1 transformed; covariate), sex (factor) and all possible three-way and two-way interactions between noise, light and sex as explanatory variables. We used interactions with “sex” as there may be sex-specific differences in physiology (e.g. oxidative status), growth rate^46, 47^ and environmental sensitivity (reviewed in ref. 48). For the models on Hp and NOx, we additionally included fledging mass, bleeding time and weather condition as covariates. Time of day and temperature might influence Hp and NOx levels^25, 26^ and body mass is a measure of condition and may therefore be related to physiological measurements. Data on weather conditions (daily average rain in mm, wind speed in km/h and temperature in °C) were obtained for the day of sampling from a local meteorological station in Antwerp. These weather data were used in a Principal Component Analysis (PCA) to obtain an overall variable for weather condition which explained 54.5% of the variance for temperature, rain and wind.

We furthermore also constructed alternative models where noise and light (and their interactions) were replaced by either distance to the nearest road or distance to the highway (covariates). Distance to road/highway was not used in combination with noise and light in one model in order to prevent collinearity and overfitting the model. Distance to the nearest road and distance to the highway were correlated with each other (Spearman rank *r* = 0.3, *p* = 0.02, *N* = 85). Distance to the nearest road/highway were also correlated with noise (Spearman rank *r* = −0.44, *p* < 0.01, *N* = 85; *r* = −0.57, *p* < 0.01, *N* = 85) but not to light levels (Spearman rank *r* ≤ −0.14, *p* ≥ 0.23, *N* = 85). To meet model assumptions, both Hp and NOx were square root transformed.

Finally, all models were compared (per dependent parameter) using a model selection approach based on Akaike’s information criterion for small sample sizes (MuMIn package^49^, AICc^50^). We used all models within ∆ AICc < 2 of the top model to obtain model-averaged estimates and standard errors for each explanatory variable and relative variable importance is calculated (MuMIn package^49, 50^). Models within ∆ AICc < 2 have substantial support or evidence^51^.

## Results

### Fledging mass was unrelated to anthropogenic noise and/or light

Noise, the interaction between noise and light, and distance to the road/highway received no support in the fledging mass models (Table 3). The top ranked model included only the variable sex, with male nestlings being heavier than female nestlings (15.8 ± 0.2 g and 15.2 ± 0.2 g, respectively). The model ranked second additionally included light (∆ AICc = 1; there were no other models with ∆ AICc < 2 of the top model). However, light did not contribute substantially to variation in fledging mass (*β* = 0.08 ± 0.67 g).

**Table 3:** Results of the fledging mass, Hp and NOx model selection procedure based on AICc.

	AICc	∆ AICc	Akaike weight
**Fledging mass model**			
s	1834.9	0	0.622
light + s	1835.9	1	0.378
**Haptoglobin model**			
b + ld + s + noise + light + light:s	2651.3	0.00	0.093
b + s + light + noise + light:s + noise: light	2651.3	0.07	0.090
b + ld + s + light + noise + light:s + noise: light	2651.6	0.38	0.077
ld + s + light + noise + light:s	2651.7	0.44	0.074
b + s + light + noise + light:s	2651.9	0.60	0.069
b + ld + s + light + noise	2651.9	0.60	0.069
b + ld + s + noise	2652.1	0.81	0.062
b + s + light + noise + noise: light	2652.3	1.06	0.055
ld + s + light + noise	2652.4	1.11	0.053
b + s + light + noise	2652.4	1.18	0.052
b + ld + light + noise	2652.6	1.35	0.047
b + ld + s + light + noise + noise: light	2652.6	1.38	0.047
ld + s + noise	2652.7	1.44	0.045
b + s + noise	2652.7	1.47	0.045
ld + s + light + noise + light:s + noise: light	2652.8	1.50	0.044
b + ld + noise	2652.8	1.52	0.044
ld + light + noise	2653.2	1.93	0.035
**Nitric oxide model**			
fledging mass	612.8	0	0.73
fledging mass + s	614.8	1.99	0.27

Linear mixed models with “NestID” as random factor were used to avoid pseudoreplication. Top ranked models included noise in combination with sex (s), light, brood size (b), laying date (ld), fledging mass, the interaction light:sex (light:s) and/or the interaction noise:light. Fledging mass models were run on data from 562 nestlings from 85 nests; Hp models on data from 475 nestlings from 78 nests and NOx models on data from 344 nestlings from 58 nests. Only models within ∆ AICc < 2 of the top model are shown.

### Haptoglobin, nitric oxide and relationships with anthropogenic noise and/or light

**Figure 1:**
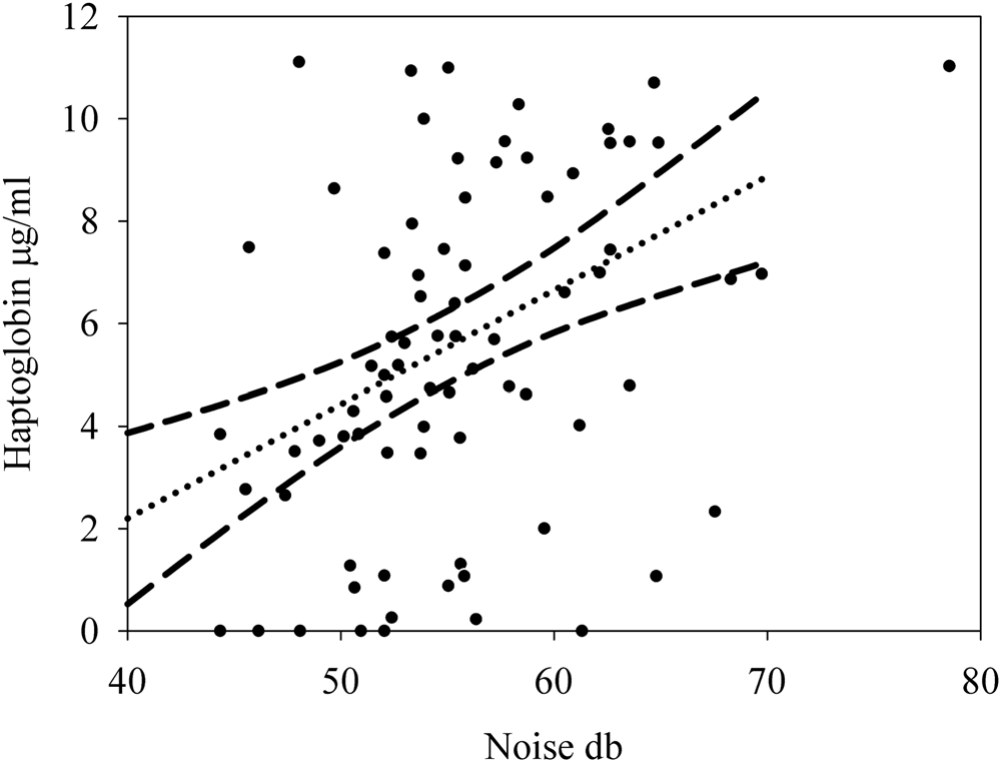
Nestlings exposed to higher levels of noise had higher levels of haptoglobin. Raw data of average Hp concentration per nest (square root transformed) in response to measured noise levels at the nest entrance. Lines represent model-averaged estimate and 95% confidence intervals (see *Statistical analysis* and Tables 2 and 3; partial *R* ^*2*^ = 0.116), based on data from 475 nestlings of 78 nests.

The interaction of noise and light received support in the Hp models but did not contribute substantially in explaining variation in Hp (Tables 3 and 4). Distance to the road/highway received no support in the Hp models (Table 3). All supported models to explain variation in Hp (within ∆ AICc < 2 of the top model) contained noise as an explanatory covariate, and nestlings exposed to higher noise levels had higher Hp levels (*β* = 0.20 ± 0.06 µg/ml Hp square root transformed; Fig. 1 and Tables 3 and 4). Light was also included in some of the supported models, but based on its estimated effect it did not contribute substantially to Hp variation (*β* = −8.54 ± 12.47 µg/ml Hp square root transformed; Tables 3 and 4).

**Table 4:** Results from the Hp model selection procedure showing parameter estimates and selection probabilities (see *Statistical analysis* and Table 2).

Parameter	Estimate ± SE	RVI	Effect size *R* ^*2*^
Brood size	0.46 ± 0.20	0.75	0.059
Laying date	−0.12 ± 0.04	0.69	0.072
Sex	−0.69 ± 0.38	0.87	0.008
Light	−8.54 ± 12.47	0.80	0.044
Noise	0.20 ± 0.06	1.00	0.116
Light:Sex	0.83 ± 1.41	0.45	0.001
Noise:Light	0.35 ± 0.19	0.31	0.043

Only factors that were used for model averaging are shown. Models were run on data from 475 nestlings of 78 nests. Haptoglobin levels had been square root transformed. Relative variable importance (RVI) are shown as well as effect sizes (partial *R* ^*2*^’s) which were calculated following Edwards, *et al*.^74^.

Nitric oxide models including anthropogenic effects as explanatory variables (light, noise, and distance to the road/highway) received no support (Table 3). The top ranked models for nitric oxide contained fledging mass and sex as explanatory variables (there was only one model within ∆ AICc < 2 of the top model). Heavier nestlings had lower levels of NOx (*β* = −0.096 ± 0.022 µmol/l square root transformed) and males tended to have higher levels of NOx (*β* = 0.12 ± 0.06 µmol/l square root transformed).

## Discussion

In this study we show that ambient anthropogenic noise was associated with the physiology of free-living great tit nestlings. However, noise, light, and their interaction were unrelated to fledging mass. Noise exposure did explain a significant part of the variation in nestling haptoglobin (Hp) concentration but not in nitric oxide (NOx) concentration. Light was unrelated to Hp and NOx. Against our expectations, we found no additive or synergistic effect of noise and light on nestling physiology. Distance of the nest to the road or highway was also unrelated to nestling fledging mass and physiology (Hp and NOx).

Nestlings exposed to higher levels of noise pollution had higher concentrations of Hp. Distance to the road/highway was not associated with concentration of Hp, while it was correlated with noise levels. The association of noise with Hp seems therefore to be independent from other potential confounding variables related to proximity to a road. Haptoglobin has besides anti-inflammatory also antioxidative properties^52^. Noise pollution may increase oxidative stress^53^ which may have led to the increase in Hp as part of a compensatory mechanism^54^. Alternatively, noise exposure may lead to stress (increased corticosterone levels^55^). Stress is known to affect baseline innate immunity^56, 57^ and may therefore lead to elevated Hp concentrations.

Whether elevated Hp levels at higher noise exposure have long-term fitness consequences is still unclear. Nonetheless, increasing levels of Hp are potentially energy demanding and trade-offs could occur with life-history traits, such as survival or life-time reproduction. In frigatebird nestlings (*Fregata magnificens*) facing a herpesvirus outbreak, plasma concentrations of Hp were predictive for survival^58^. While innate immunity has been linked to long-term survival (e.g. refs 23 and 59), Hp concentrations in great tit nestlings were not found to be predictive of local recruitment^41^. However, noise has been shown to have a negative effect on great tit reproduction^38^ and to reduce telomere length in nestling house sparrows^12^. Whether elevated levels of Hp which we found here contribute to these negative effects on reproduction and/or survival remains to be examined.

Despite that noise exposure was related to Hp concentrations, neither the combination of noise and light exposure nor light exposure itself were related to Hp. Moreover, noise and light exposure were unrelated to fledging mass and NOx and there was no combined effect. Nestlings inside nest boxes may be exposed to only limited amounts of artificial light which might to an extent explain why we found no effect of light pollution on their physiology. Nonetheless, indirect effects might occur as adult great tits may be affected and, for example, show disrupted activity patterns^29, 60^, altered foraging behaviour^61^, increased stress levels^62^ or advanced laying dates^63^. Interestingly, although noise did not affect daily timing of dawn song^64^, light pollution did appear to affect song behaviour in great tits^65–67^ (but see also ref. 68). These studies indicate that in adult free-living great tits, activity patterns and perhaps foraging behaviour and subsequently nestlings could be (indirectly) affected by light pollution while noise pollution might cause more direct physiological effects in nestlings.

Low light intensities could have been expected to lead to direct physiological changes. Light intensity measured at the nest boxes in our population ranged between 0.01 and 6.4 lux (0.01 lux is the lower limit of the light meter). In experimental studies, sleep behaviour of adult great tits and nightly activity and physiology of nestlings (fledging mass, Hp and NOx) were affected by light intensities of 1.6 (adult sleep behaviour and nestling nightly activity, refs 29 and 69) and 3.0 lux (nestling physiology^14, 15^). However, a low light intensity of 0.3 lux was already sufficient to advance reproductive physiology and decrease melatonin levels of adult male blackbirds (*Turdus merula*)^70, 71^ and even lower light intensities of 0.05 lux affect nightly activity in adult male great tits^60^. Very low levels of light exposure could thus potentially still have caused physiological effects, especially in combination with exposure to noise pollution. Nonetheless, despite the potentially low light intensities to which our nestlings were exposed, our results are still relevant for cavity-nesting species such as great tits where it would appear that light pollution would have a limited direct effect on nestling development and physiology.

Although we are one of the first to examine possible additive or synergetic effects of ambient noise and light pollution on free-living developing animals, our study comes with some limitations. First, we studied how ambient levels of light pollution may affect nestling physiology, however, we cannot know the exact light levels to which nestlings were exposed. Females usually sleep inside the nest box (even when nestlings are 15 days old) and to an extent also on top of the nestlings^29^ which may limit the amount of light exposure of the nestlings and also severely complicates taking light measurements at the level of the nestlings. Experiments are therefore necessary to examine direct and indirect effects of artificial light at night. However, our study does represent a natural situation for cavity nesting species exposed to light pollution. Second, another limitation of our study is its inherently correlational nature which may make it difficult (or impossible) to prove causation compared to experimental studies. For example, in the current study we took measurements of Hp and NOx at day 15, while in our experimental studies we took measurements at 13 days after hatching and again after a two night exposure to experimental artificial light inside the nest box^14, 15^. Such an experimental design (within-individual design with an additional control group) is very powerful to detect possible differences caused by our treatment. Moreover, under natural conditions such as in our current study, the variation in Hp and NOx (amongst other physiological markers) among nestlings of the same nest is higher within the same nest than among nests^40^. This might increase the difficulty of detecting an effect of pollutants which occurs at the nest level^40^. Here we have found that noise was associated with higher levels of Hp. Direct effects of noise on nestling physiology have been reported by Meillère, *et al*.^12^ who showed that experimental exposure to traffic noise reduced telomere length in nestling house sparrows, although growth and fledging success was unaffected. In tree frogs (*Hyla arborea*) noise also had a direct effect, increasing stress hormones and inducing an immunosuppressive effect^72^. There is also evidence that air pollution from roads could affect nestlings^73^. However, our results appear to suggest that it is the noise from the roads and not air pollution that affected nestling physiology as models including proximity to the road/highway as an explanatory variable had no support. However, whether there is a causal relationship between noise and higher levels of Hp needs to be examined further with experimental studies. Third, here we used nestlings from a cavity-nesting bird as a model species, the great tit, because they readily accept nest boxes to breed. Although these results are perhaps difficult to extend to open-nesting species, a similar study on this scale (more than 500 nestlings were included in the current study) using nestlings of open-nesting birds is much more difficult. However, such nestlings might be exposed to similar or higher levels of noise and light pollution and may experience an additive or synergistic effect of these pollutants, which remains to be studied.

In conclusion, this study demonstrates that, contrary to our expectations, there was no additive or synergistic effect of ambient noise and light on nestling physiology or fledging mass. Anthropogenic noise but not light was associated with the physiology of 15 day old nestlings from a cavity-nesting species. This could have long lasting adverse consequences. Our study on free-living nestlings complements experimental studies^12, 13^ and suggests that the urban environment may entail important costs for developing animals.

## References

[1] Barber JR, Crooks KR, Fristrup KM (2010). The costs of chronic noise exposure for terrestrial organisms. Trends Ecol. Evol..

[2] Davies TW, Duffy JP, Bennie J, Gaston KJ (2014). The nature, extent, and ecological implications of marine light pollution. Frontiers in Ecology and the Environment.

[3] Swaddle JP (2015). A framework to assess evolutionary responses to anthropogenic light and sound. Trends Ecol. Evol..

[4] Falchi F (2016). The new world atlas of artificial night sky brightness. Science Advances.

[5] Hölker, F. *et al*. The dark side of light: a transdisciplinary research agenda for light pollution policy. *Ecol*. *Soc*. **15** (2010).

[6] Hölker F, Wolter C, Perkin EK, Tockner K (2010). Light pollution as a biodiversity threat. Trends Ecol. Evol..

[7] Halfwerk, W. & Slabbekoorn, H. Pollution going multimodal: the complex impact of the human-altered sensory environment on animal perception and performance. *Biol*. *Lett*. **11**, doi:10.1098/rsbl.2014.1051 (2015). 10.1098/rsbl.2014.1051 PMC4424613 25904319

[8] Isaksson C (2015). Urbanization, oxidative stress and inflammation: a question of evolving, acclimatizing or coping with urban environmental stress. Funct. Ecol..

[9] McMahon, T. A., Rohr, J. R. & Bernal, X. E. Light and noise pollution interact to disrupt interspecific interactions. *Ecology*, **98**, 1290-1299, doi:10.1002/ecy.1770 (2017). 10.1002/ecy.1770 PMC7183140 28170099

[10] Gaston, K. J., Visser, M. E. & Holker, F. The biological impacts of artificial light at night: the research challenge. *Philos*. *Trans*. *R*. *Soc*. *Lond*. *B*. *Biol*. *Sci*. **370**, doi:10.1098/rstb.2014.0133 (2015). 10.1098/rstb.2014.0133 PMC4375372 25780244

[11] Spoelstra, K. *et al*. Experimental illumination of natural habitat–an experimental set-up to assess the direct and indirect ecological consequences of artificial light of different spectral composition. *Philos*. *Trans*. *R*. *Soc*. *Lond*. *B*. *Biol*. *Sci*. **370**, doi:10.1098/rstb.2014.0129 (2015). 10.1098/rstb.2014.0129 PMC4375369 25780241

[12] Meillère A, Brischoux F, Ribout C, Angelier F (2015). Traffic noise exposure affects telomere length in nestling house sparrows. Biol. Lett..

[13] Salmon, P., Nilsson, J. F., Nord, A., Bensch, S. & Isaksson, C. Urban environment shortens telomere length in nestling great tits, *Parus major*. *Biol*. *Lett*. **12**, doi:10.1098/rsbl.2016.0155 (2016). 10.1098/rsbl.2016.0155 PMC4938044 27303051

[14] Raap T, Casasole G, Pinxten R, Eens M (2016). Early life exposure to artificial light at night affects the physiological condition: An experimental study on the ecophysiology of free-living nestling songbirds. Environ. Pollut..

[15] Raap T (2016). Artificial light at night affects body mass but not oxidative status in free-living nestling songbirds: an experimental study. Sci Rep.

[16] Monaghan P (2008). Early growth conditions, phenotypic development and environmental change. Philos. Trans. R. Soc. Lond. B. Biol. Sci..

[17] Francis CD, Barber JR (2013). A framework for understanding noise impacts on wildlife: an urgent conservation priority. Frontiers in Ecology and the Environment.

[18] Blickley JL (2012). Experimental chronic noise is related to elevated fecal corticosteroid metabolites in lekking male greater sage-grouse (*Centrocercus urophasianus*). PLoS ONE.

[19] Kight CR, Swaddle JP (2011). How and why environmental noise impacts animals: an integrative, mechanistic review. Ecol. Lett..

[20] Fonken LK, Nelson RJ (2016). Effects of light exposure at night during development. Curr Opin Behav Sci.

[21] Peig J, Green AJ (2009). New perspectives for estimating body condition from mass/length data: the scaled mass index as an alternative method. Oikos.

[22] Maness TJ, Anderson DJ (2013). Predictors of juvenile survival in birds. Ornithological Monographs.

[23] Bowers EK (2014). Neonatal body condition, immune responsiveness, and hematocrit predict longevity in a wild bird population. Ecology.

[24] Both C, Visser ME, Verboven N (1999). Density-dependent recruitment rates in great tits: the importance of being heavier. Proc. Biol. Sci.

[25] Matson KD, Horrocks NP, Versteegh MA, Tieleman BI (2012). Baseline haptoglobin concentrations are repeatable and predictive of certain aspects of a subsequent experimentally-induced inflammatory response. Comp. Biochem. Physiol. A Mol. Integr. Physiol..

[26] Sild E, Horak P (2009). Nitric oxide production: an easily measurable condition index for vertebrates. Behav. Ecol. Sociobiol..

[27] Stracey CM, Wynn B, Robinson SK (2014). Light pollution allows the northern mockingbird (Mimus polyglottos) to feed nestlings after dark. Wilson J Ornithol.

[28] Quinn JL, Whittingham MJ, Butler SJ, Cresswell W (2006). Noise, predation risk compensation and vigilance in the chaffinch *Fringilla coelebs*. J. Avian Biol..

[29] Raap T, Pinxten R, Eens M (2016). Artificial light at night disrupts sleep in female great tits (*Parus major*) during the nestling period, and is followed by a sleep rebound. Environ. Pollut..

[30] Lucass C, Eens M, Muller W (2016). When ambient noise impairs parent-offspring communication. Environ. Pollut..

[31] Crino OL, Van Oorschot BK, Johnson EE, Malisch JL, Breuner CW (2011). Proximity to a high traffic road: glucocorticoid and life history consequences for nestling white-crowned sparrows. Gen. Comp. Endocrinol..

[32] Fahrig L, Rytwinski T (2009). Effects of roads on animal abundance: an empirical review and synthesis. Ecol. Soc..

[33] Van Duyse E, Pinxten R, Snoeijs T, Eens M (2005). Simultaneous treatment with an aromatase inhibitor and an anti-androgen decreases the likelihood of dawn song in free-living male great tits. Parus major. Horm. Behav..

[34] Rivera-Gutierrez HF, Pinxten R, Eens M (2012). Tuning and fading voices in songbirds: age-dependent changes in two acoustic traits across the life span. Anim. Behav..

[35] Rivera-Gutierrez HF, Pinxten R, Eens M (2010). Multiple signals for multiple messages: great tit, *Parus major*, song signals age and survival. Anim. Behav..

[36] Vermeulen A, Muller W, Eens M (2016). Vitally important - does early innate immunity predict recruitment and adult innate immunity?. Ecol Evol.

[37] Van Duyse E, Pinxten R, Eens M (2000). Does testosterone affect the trade-off between investment in sexual/territorial behaviour and parental care in male great tits?. Behaviour.

[38] Halfwerk W, Holleman LJM, Lessells CM, Slabbekoorn H (2011). Negative impact of traffic noise on avian reproductive success. J. Appl. Ecol..

[39] Departement Leefmilieu Natuur en Energie. Strategische geluids-belastingkaart volgens de Europese richtlijn 2002/49/EG voor wegen met meer dan 3 miljoen voertuigen per jaar en aanvullende wegen, https://www.lne.be/geluidsmeetnet-cijfers-en-rapporten (2016)(Date of access 1/11/2016).

[40] Vermeulen A (2015). Sources of variation in innate immunity in great tit nestlings living along a metal pollution gradient: an individual-based approach. Sci. Total Environ..

[41] Vermeulen A, Eens M, Zaid E, Müller W (2016). Baseline innate immunity does not affect the response to an immune challenge in female great tits (*Parus major*). Behav. Ecol. Sociobiol..

[42] Griffiths R, Double MC, Orr K, Dawson RJG (1998). A DNA test to sex most birds. Mol. Ecol..

[43] R: A language and environment for statistical computing v. 3.3.2 (R Foundation for Statistical Computing, Vianna, Austria, 2016).

[44] Zuur, A. F., Ieno, E. N., Walker, N. J., Saveliev, A. A. & Smith, G. M. *Mixed effects models and extensions in ecology with R*. (Springer, 2009).

[45] Bates D, Machler M, Bolker BM, Walker SC (2015). Fitting Linear Mixed-Effects Models Using lme4. Journal of Statistical Software.

[46] Giordano M, Costantini D, Tschirren B (2015). Sex-specific effects of prenatal and postnatal nutritional conditions on the oxidative status of great tit nestlings. Oecologia.

[47] Speakman JR (2015). Oxidative stress and life histories: unresolved issues and current needs. Ecol Evol.

[48] Jones KS, Nakagawa S, Sheldon BC (2009). Environmental sensitivity in relation to size and sex in birds: meta-regression analysis. Am. Nat..

[49] Barton, K. MuMIn Multi-model inference v. 1.15.6 (2016).

[50] Anderson DR, Burnham KP (2002). Avoiding pitfalls when using information-theoretic methods. J. Wildl. Manag.

[51] Burnham KP, Anderson DR (2004). Multimodel Inference: Understanding AIC and BIC in Model Selection. Sociological Methods & Research.

[52] Jelena A (2013). Haptoglobin and the inflammatory and oxidative status in experimental diabetic rats: antioxidant role of haptoglobin. J. Physiol. Biochem..

[53] Cheng L, Wang SH, Chen QC, Liao XM (2011). Moderate noise induced cognition impairment of mice and its underlying mechanisms. Physiol. Behav..

[54] Costantini D, Verhulst S (2009). Does high antioxidant capacity indicate low oxidative stress?. Funct. Ecol..

[55] Bonier F (2012). Hormones in the city: endocrine ecology of urban birds. Horm. Behav..

[56] Matson KD, Tieleman BI, Klasing KC (2006). Capture stress and the bactericidal competence of blood and plasma in five species of tropical birds. Physiol. Biochem. Zool..

[57] Martin LB (2009). Stress and immunity in wild vertebrates: timing is everything. Gen. Comp. Endocrinol..

[58] Sebastiano M (2017). Corticosterone, inflammation, immune status and telomere length in frigatebird nestlings facing a severe herpesvirus infection. Conserv Physiol.

[59] Cichon M, Dubiec A (2005). Cell-mediated immunity predicts the probability of local recruitment in nestling blue tits. J. Evol. Biol.

[60] de Jong M (2016). Dose-dependent responses of avian daily rhythms to artificial light at night. Physiol. Behav..

[61] Titulaer M, Spoelstra K, Lange CY, Visser ME (2012). Activity patterns during food provisioning are affected by artificial light in free living great tits (Parus major). PLoS ONE.

[62] Ouyang, J. Q. *et al*. Stressful colours: corticosterone concentrations in a free-living songbird vary with the spectral composition of experimental illumination. *Biol*. *Lett*. **11**, doi:10.1098/rsbl.2015.0517 (2015). 10.1098/rsbl.2015.0517 PMC4571683 26311159

[63] de Jong, M. *et al*. Effects of nocturnal illumination on life-history decisions and fitness in two wild songbird species. *Philos*. *Trans*. *R*. *Soc*. *Lond*. *B*. *Biol*. *Sci*. **370**, doi:10.1098/rstb.2014.0128 (2015). 10.1098/rstb.2014.0128 PMC4375368 25780240

[64] Da Silva A, Samplonius JM, Schlicht E, Valcu M, Kempenaers B (2014). Artificial night lighting rather than traffic noise affects the daily timing of dawn and dusk singing in common European songbirds. Behav. Ecol..

[65] Kempenaers B, Borgstrom P, Loes P, Schlicht E, Valcu M (2010). Artificial night lighting affects dawn song, extra-pair siring success, and lay date in songbirds. Curr. Biol..

[66] Da Silva A, Valcu M, Kempenaers B (2015). Light pollution alters the phenology of dawn and dusk singing in common European songbirds. Philos. Trans. R. Soc. Lond. B. Biol. Sci..

[67] Da Silva A, Valcu M, Kempenaers B (2016). Behavioural plasticity in the onset of dawn song under intermittent experimental night lighting. Anim. Behav..

[68] Da Silva A (2017). Experimental illumination of a forest: no effects of lights of different colours on the onset of the dawn chorus in songbirds. Royal Society Open Science.

[69] Raap T, Pinxten R, Eens M (2015). Light pollution disrupts sleep in free-living animals. Sci Rep.

[70] Dominoni DM, Goymann W, Helm B, Partecke J (2013). Urban-like night illumination reduces melatonin release in European blackbirds (*Turdus merula*): implications of city life for biological time-keeping of songbirds. Frontiers in Zoology.

[71] Dominoni D, Quetting M, Partecke J (2013). Artificial light at night advances avian reproductive physiology. Proc. Biol. Sci.

[72] Troianowski, M., Mondy, N., Dumet, A., Arcanjo, C. & Lengagne, T. Effects of traffic noise on tree frog stress levels, immunity and color signaling. *Conserv*. *Biol*., n/a–n/a, doi:10.1111/cobi.12893 (2017). 10.1111/cobi.12893 28074559

[73] Peach WJ, Vincent KE, Fowler JA, Grice PV (2008). Reproductive success of house sparrows along an urban gradient. Anim. Conserv..

[74] Edwards LJ, Muller KE, Wolfinger RD, Qaqish BF, Schabenberger O (2008). An R^2^ statistic for fixed effects in the linear mixed model. Stat. Med..

